# Hypoxic Isolated Abdominal Perfusion (HAP) chemotherapy for non-operable advanced staged ovarian cancer with peritoneal carcinosis: an experience in 45 platinum-refractory ovarian cancer patients

**DOI:** 10.1007/s13193-019-00922-9

**Published:** 2019-04-16

**Authors:** Karl Reinhard Aigner, Emir Selak, Sabine Gailhofer, Kornelia Aigner

**Affiliations:** Department of Surgical Oncology, Medias Klinikum GmbH & Co KG, Krankenhausstr. 3a, 84489 Burghausen, Germany

**Keywords:** Ovarian cancer, Quality of life, Chemoresistance, Isolated abdominal perfusion, Intra-arterial chemotherapy

## Abstract

In order to break through drug resistance in platinum-refractory ovarian cancer, augmented drug exposure was administered to the abdomen by means of an isolated perfusion system. Four cycles of isolated hypoxic abdominal perfusion with cisplatin, adriamycin, and mitomycin were conducted in 4-week intervals. Cisplatin and adriamycin were chosen because of their increased cytotoxicity under hypoxic conditions. Chemofiltration was performed for prophylaxis of cumulative toxicity of adriamycin and mitomycin. The study included 45 patients with recurrent epithelial ovarian cancer who had prior platinum containing therapies (3, stage Federation of Gynecology and Obstetrics (FIGO) IIIB; 20, stage FIGO IIIC; 22; stage FIGO IV). The median survival rate in stage FIGO IIIBC was 12 months, and in stage IV was 10 months. The tumor marker decreased to complete response or partial response at 17.8% and 55.6% of the patients. CT or MRI visualization showed complete response in 4.1%, and partial response was in 54.1%. Complete resolution of ascites was noted in 30% of cases and substantial reduction in another 43%. Toxicity was generally low. Quality of life was improved in the majority of cases. Bone-marrow suppression ranged between WHO grade 1 and 2, and in patients with previous third- or fourth-line chemotherapy, it was WHO grade 3. Isolated hypoxic abdominal perfusion with chemofiltration for patients with progressive and platinum-refractory stage III and IV ovarian cancer is an effective therapy, breaking through chemoresistance and offering comparably long survival at good quality of life.

## Background

Ovarian cancer is the leading cause of death among all gynecological cancers. The standard therapy option is still complete cytoreduction when possible, combined with chemotherapy [1–3]. Despite initial response rates of 70–80% to platinum-based combination chemotherapies with taxanes, platinum-resistant recurrences occur very frequently within 2 years. The shorter the recurrence-free interval, the lower the prospect of a renewed response to chemotherapy [4–6]. While increased drug exposure could theoretically lead to a renewed response, they are not universally administered due to increased toxicity [7–15]. Even high-dose or modified combination therapy did not result in any real progress [16–19]. Only recently two randomized studies showed that the addition of HIPEC to cytoreductive surgery resulted in longer recurrence-free and overall survival than cytoreductive surgery alone [20, 21].

Alternatively, consideration may be given to new drugs or targeted substances [22–24]. As the response behavior rises sharply with an increased dose or concentration of active agents, we surmised that an isolated extracorporeal perfusion would increase local exposure and break through any existing cytostatic resistance. In order to limit systemic toxicity, which could adversely affect quality of life, chemofiltration for detoxification was carried out, following the isolated perfusion procedure [26, 27]. A case series of 45 heavily pretreated patients with ovarian cancer treated with hypoxic abdominal perfusion is herein reported. It has been applied in patients with advanced and recurrent tumors, resistant to platinum-containing combination therapies.

## Methods

### Patients

The study included 45 patients at the clinical stages of Federation of Gynecology and Obstetrics (FIGO) IIIB, IIIC, and IV (*n* = 3, 20, and 22, respectively) treated in one institution between 2006 and 2017. All patients were progressive after systemic chemotherapy and had received at least two lines of platinum-containing combinations; three had undergone third-line and one patient fourth-line therapies. Previously given drugs contained combinations of cisplatin or carboplatin with paclitaxel, caelyx, treosulfan, or bevacizumab. The minimum chemotherapy-free interval before the start of abdominal perfusion therapy was 4 weeks. Performance status was ECOG 1 (7 patients), ECOG 2 (13 patients), and ECOG 3 (25 patients). Forty one patients (91%) had peritoneal carcinosis and according to MRI, four patients had no evidence of peritoneal carcinosis. Progression of peritoneal carcinosis was classified by the affection of either two quadrants (11 patients, 24%) or four quadrants (30 patients, 67%). Seventeen patients had additional liver metastases, and three patients had metastases on the spleen. Lymphatic metastases were detected in 13 patients; one of them was inguinal, and in one case was mediastinal. Median number of metastatic locations (peritoneum considered as one location) was two. Surgery as a primary measure after progression was considered as unfeasible in all cases.

Investigations were performed in compliance with the principles of good clinical practice outlined in the Declaration of Helsinki and federal guidelines, and had approval by the Institutional Review Committee. Informed consent was obtained from each participant or participant’s guardian. Patients were required to be > 18 years of age and have an ECOG performance status ≤ 3. Exclusion criteria included cardiovascular diseases, as well as uncontrolled diabetes or serious infectious diseases. The leucocyte count had to be no less than 2500/μl, but more importantly should not show a declining trend before start of the therapy. The same applied to the thrombocyte count, with a threshold of no less than 100,000/μl.

### Isolated Hypoxic Abdominal Perfusion

**Fig. 1:**
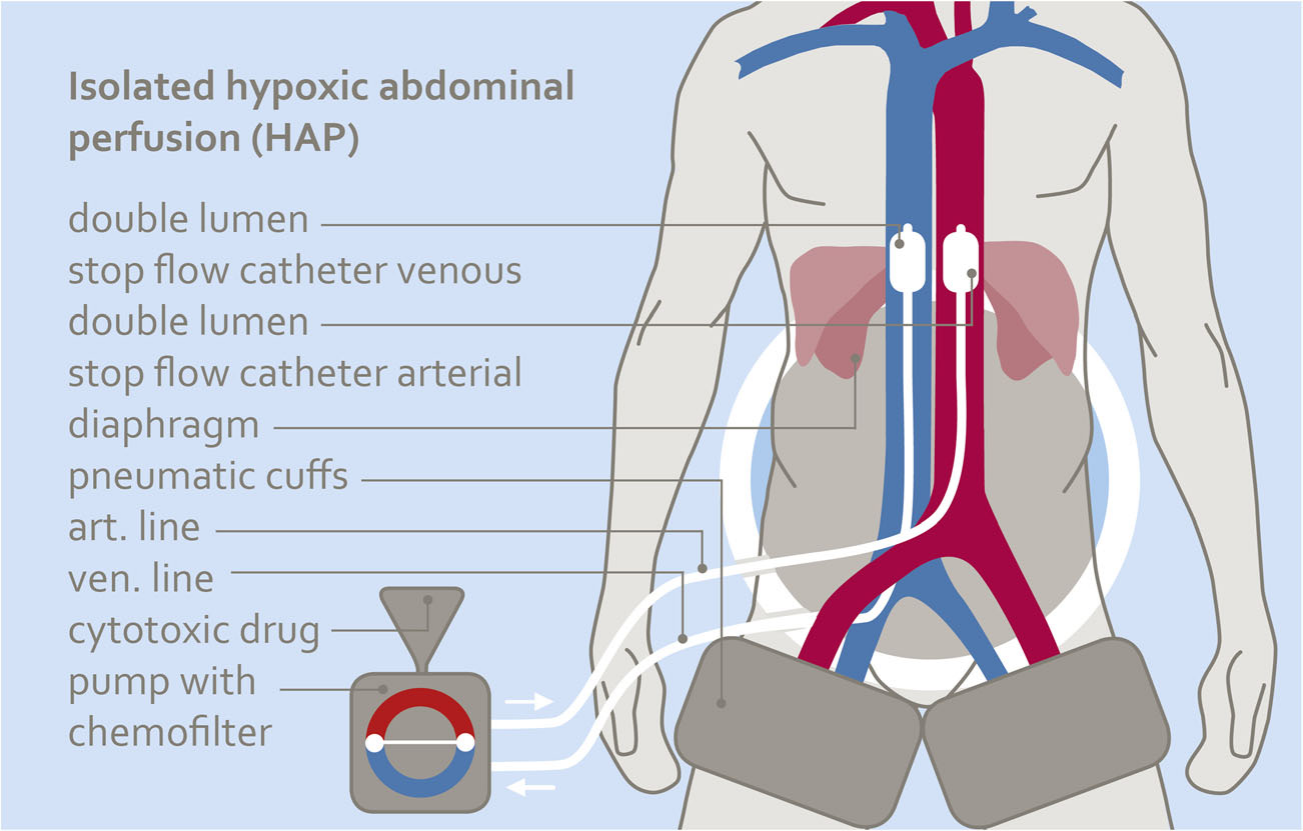
Isolated hypoxic abdominal perfusion via a femoral access. The balloon catheters are positioned beneath the diaphragm and connected with an extracorporeal roller pump

Under general anesthetic, the arteria and vena femoralis were exposed and tied with tourniquets. An arterial stop-flow catheter (PfM, Cologne, Germany and Dispomedica, Hamburg, Germany) was placed over the arterial transverse incision under X-ray monitoring, with the balloon above the diaphragm. The venous stop-flow catheter was secured with a purse-string suture and inserted through a stab incision. The balloon was placed in the inferior vena cava above the confluence of the right hepatic vein and below the right atrium. After blocking the balloons and checking the position with contrast medium, the balloons were unblocked again and both thighs were initially blocked with pneumatic cuffs. Under temporary hyperoxygenation, the chemotherapeutic agents were then applied through the aortal catheter as a bolus and both balloon catheters were immediately blocked again (Fig. 1). It is mandatory to inject the drugs under prior hyperoxygenation directly before balloon-blocking. The subsequent therapy was conducted for 15 min under hypoxic conditions in which mitomycin and adriamycin unlike other chemotherapeutics produce increased tumor toxicity [28]. Only the effect of cisplatin is not influenced by the pH value. Leakage monitoring of the isolated abdominal perfusion segment was not required; as after 15 min of hypoxic perfusion, the stop-flow balloon catheters and femoral pressure cuffs were unblocked and the systemic chemofiltration started immediately. Chemofiltration is maintained up to a substitution volume of at least 4 l of filtrate. After completing chemofiltration, the catheters were removed and the artery and vein sutured successively.

### Treatment and Drug Regimen

The isolated abdominal hypoxic perfusion was conducted in 4 cycles at 4-week intervals. The cytostatic drugs used were cisplatin, adriamycin, and mitomycin. Using a bolus injection in the abdominal aorta at the diaphragm level, the maximum intra-arterial total dose of CDDP was 70 mg at one shot. For ADM, the maximum dose was 50 mg and for MMC 20 mg at one shot. For a patient of 70 ± 5 kg, the overall dose of CDDP was 60 mg, ADM 40 mg, and MMC 20 mg. Due to their intra-aortal administration, the cytostatic drugs were not dosed by body weight for extremely obese patients.

Leucocytes and thrombocytes were monitored weekly after every treatment; while decreasing close to the lowest nadir, checks were carried out every 2 days until the blood count started to reemerge.

### Response Evaluation and Statistical Analysis

The main endpoint of the trial was quality of life, overall survival, and clinical response rates. Tumor responses were visualized and assessed in accordance with Response Evaluation Criteria in Solid Tumors (RECIST version 1.1) at 2 to 4 weeks after every second treatment cycle. Responses were evaluated by CT, magnetic resonance imaging (MRI), and positron emission tomography (PET). Plasma levels of the tumor marker CA 12-5 were determined before any treatment. The extent of residual disease, the degree of response in ascites, as well as the course of the tumor marker CA 12-5 were evaluated and compared. A reliable pain relief was noted if pain was controlled by < 50% analgesic administration 20 days after treatment. Adverse events were assessed according to the common terminology criteria for adverse events of the National Cancer Institute. Quality of life was evaluated by a patient questionnaire according to RCT institutional QOL approvement and compared to questionnaires regarding previous systemic chemotherapy treatments. Statistics have been calculated with 95% confidence limits, or as specified, survival times were estimated using the Kaplan–Meier product limit estimator. Survival times were stratified according to clinical variables that may affect survival, and log-rank tests were used to verify significance. Statistical analyses were performed by using the institutional research software, version 28.5.14.

### Blood Sampling for Cisplatin and Mitomycin C Plasma Concentration Measurements

A series of cisplatin (CDDP) and Mitomycin C (MMC) plasma concentration measurements have been investigated. Blood samples derive from a patient with ovarian cancer and peritoneal carcinosis during and after hypoxic abdominal perfusion with CDDP and MMC. Arterial and venous drug concentrations, originating from the tumor region, were measured at minute 1, 2, and 3, respectively, following measurements at 2-min intervals.

## Results

### Response and Survival Rates

**Fig. 2:**
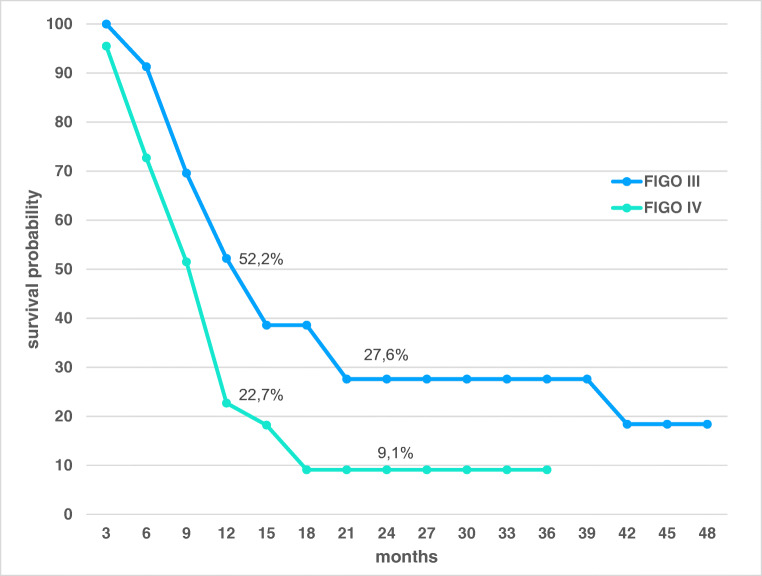
Observational survival rates of 45 advanced staged, heavily pretreated, recurrent ovarian cancer patients on hypoxic abdominal perfusion chemotherapy. Three patients were staged FIGO IIIB, 20 patients were stage IIIC, and 22 patients were staged IV. Survival times were estimated using the Kaplan–Meier product limit estimator, and follow-up for surviving patients was minimum 18 months; median follow-up was 26 months

**Fig. 3:**
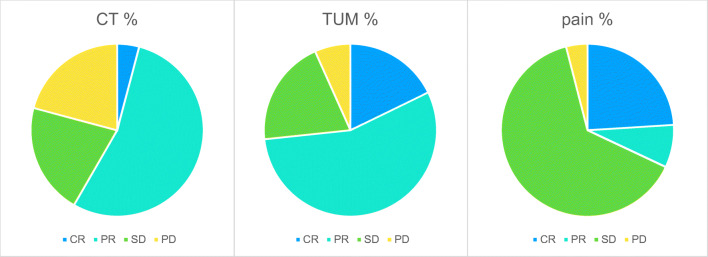
Response rates of 45 advanced staged, heavily pretreated, recurrent metastatic ovarian cancer patients on hypoxic abdominal perfusion chemotherapy. Response classification was complete response (CR), partial response (PR), stable disease (SD), and progressive disease (PD). Responses of a clinical benefit (CR, PR, SD) endured at least 8 weeks

The median progression-free survival (PFS) of all 45 patients was 6.9 months; the median overall survival was 11.3 months. The median survival rate of pretreated platinum-refractory patients at FIGO stage IIIB/C was 12.3 months, and at stage IV was 9.8 months (Fig. 2) (*p* < 0.05). Overall survival in FIGO III and IV together is 37.8% at 1 year, 18.3% at 2 years, stays 18.3% at 3 years, and 9.2% at 4 years. Time measurements started after completing multiple lines of standard chemotherapy, diagnosis of recurrent or progressive disease, and start treatment with hypoxic abdominal perfusion therapy. According to tumor marker 12-5, complete response and partial response have been achieved in 17.8% and 55.6% of all patients, respectively. CT or MRI visualization showed complete response and partial response in 4.1 and 54.1% of the cases (Fig. 3), respectively.

### Arterial and Venous Concentrations of CDDP and MMC

**Fig. 4:**
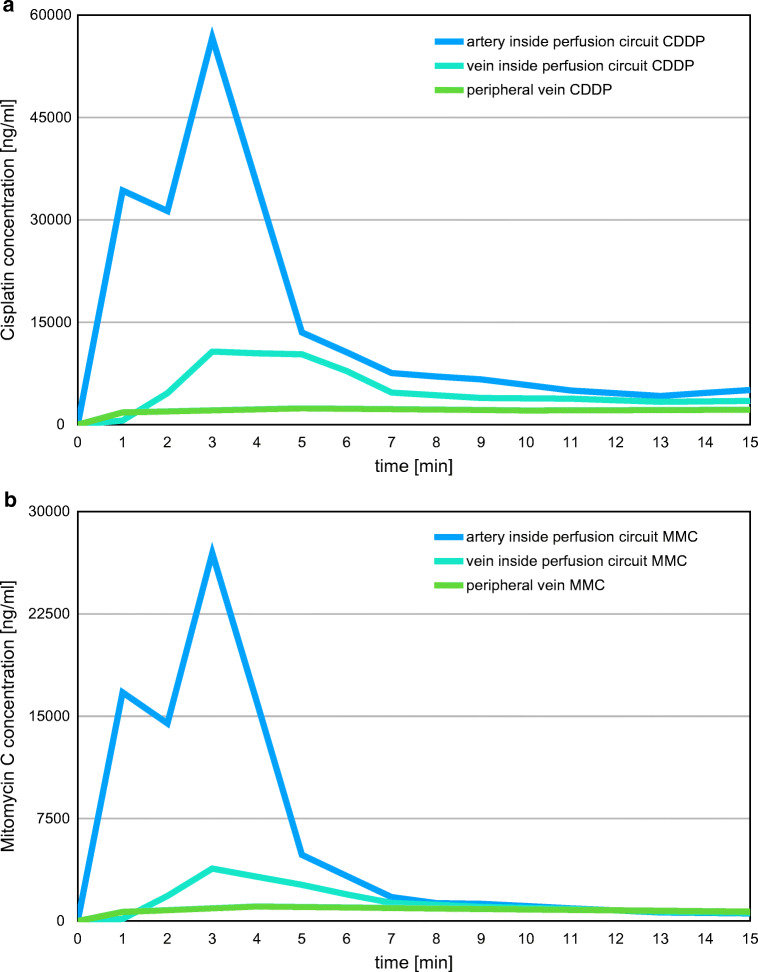
Drug concentration levels of cisplatin (**a**) and MMC (**b**) in the tumor supplying arteria (violet), the tumor draining vein (blue), and the peripheral vein (green) during and after a hypoxic abdominal perfusion. Measurements inside the perfusion circuit (arterial and venous) have been made at minute 1, 2, and 3, and then every 2 min until 35 min after drug injection. Drug levels in the peripheral vein have been measured at minute 1, 5, and after releasing of perfusion balloons at minute 15. Given drug regimen was 50 mg cisplatin and 20 mg mitomycin as a bolus injection. Both charts derive from the same perfusion event

During and after 15 min of hypoxic abdominal perfusion, drug concentrations were measured from plasma deriving from the inferior vena cava and from the aorta, both inside the perfusion area, and additionally from a peripheral vein outside the perfusion area (Fig. 4).

### Toxicity

Bone-marrow suppression was low for 43 patients and ranged between WHO grade 1 and 2. Two patients in poor general condition following third- and fourth-line chemotherapy had leucopenia and thrombocytopenia WHO grade 3. Neutropenic fever at WHO grade 4 toxicity was never observed. Fatigue syndrome never occurred before the third day post-therapy and, if it occurred, it was always associated with an increase in both LDH and the tumor marker CA 12-5. Hypodense areas seen simultaneously in CT scans were identified as an expression of post-therapeutic tumor necrosis. All of these syndromes were observed during the first week after isolated perfusion. Mild renal toxicity with slight transient elevation of creatinine was observed in seven (15.6%) patients. Neuropathy in terms of hand–foot syndrome was never observed after isolated perfusion with chemofiltration.

### Quality of Life

**Fig. 5:**
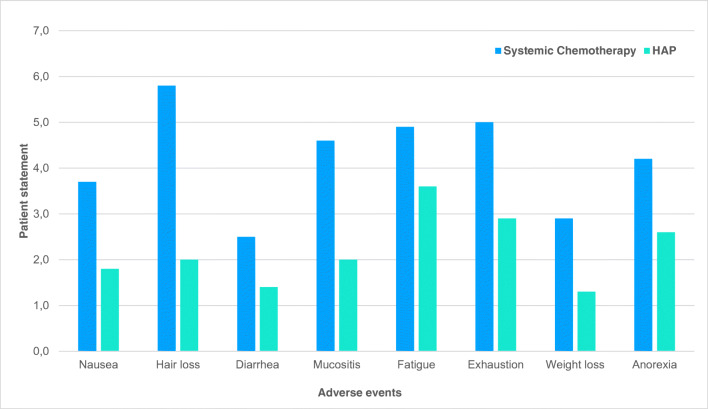
Adverse effects after systemic chemotherapy (green) and hypoxic abdominal perfusion (HAP blue). Ovarian cancer patients filled in a questionnaire about adverse effects after chemotherapy. Each possible adverse effect is scaled on a spectrum from 1 to 6 for increasing severeness of side effects. Mean reduction of adverse events was 1.86 points for hypoxic abdominal perfusion compared to systemic chemotherapy, that patients perceived at an earlier treatment period (confidence interval for 1.86 (with 91% CI 0.5)

Complete or partial response on clinical symptoms was noted among 36.3% of all patients in stages IIIB/C and IV. Stable disease was achieved in 51.1% of patients. Complete disappearance of ascites was observed in 30% of patients after only two perfusions; 43% of patients reported a substantial reduction of abdominal pressure and fluid volume and a considerable improvement in general wellbeing. As shown in Fig. 5, patients perceived hypoxic abdominal perfusion (HAP) therapy less stressful than conventional chemotherapy. A patient questionnaire showed decrease in adverse events for the symptoms nausea, hair loss, diarrhea, mucositis, fatigue, exhaustion, weight loss, and anorexia. The mean decrease over all collected data for HAP was 1.86 (confidence interval, with 91% CI 0.5). A definite decrease in pain, which was controlled by < 50% analgesic administration 20 days after treatment, is reported for 30% of patients with advanced ovarian carcinoma.

## Discussion

The limiting factor of augmented systemic drug exposure always had been the increasing toxicity such as neuropathy with hand–foot syndrome, neutropenia, or fatigue to the point of exhaustion [9–15]. Since mortality rates of ovarian cancer remain high and have hardly changed in the last three decades, more recent treatment options are being tested in the context of molecular biology research. Based on the angiogenetic properties of ovarian cancer with induction of vascularization, there has been an expectation that targeted treatments could achieve a restriction of tumor blood supply, higher response rates, and improved survival times, while sparing healthy tissue [24]. Apart from prolonging PFS in ovarian cancer, targeted antiangiogenic therapies occasionally cause severe collateral damage in terms of high blood pressure, hemorrhages, protein urea, cardiotoxicity, and gastrointestinal toxicity with spontaneous perforations [23]. In a trial on 32 patients pretreated with multiple chemotherapy regimens, positive results were achieved with bevacizumab [24]. The median survival time was 6.9 months, with a median PFS of 5.5 months. With the limitation that the patient groups are not necessarily exactly comparable, these data do not reach the median survival time of 11.3 months at a PFS of 6.9 months reported here, following hypoxic isolated abdominal perfusion.

Prolonging life while maintaining or improving quality of life should be the basic requirement for any cancer treatment. No other fundamental necessity for any treatment to be recommended should actually apply [25]. In a comparative study, we looked at patients who were in progression following systemic chemotherapy and were then treated with regional chemotherapy [29]. Patients were asked to complete a questionnaire on the severity of the most common side effects, comparing their previous systemic therapy and the regional chemotherapy. Results showed that regional chemotherapy resulted in side effects that were perceived as less severe than the ones derived from systemic chemotherapy.

Surgical tumor debulking with the aim of complete cytoreduction is intended to prolong progression-free survival even in the case of advanced diseases, but it is ultimately limited to early stages, in which the desired curative removal of all lesions is still possible [30, 31]. At clinical stage IV, there is no difference in overall survival, no matter what treatment, chemotherapy, or primary cytoreductive surgery (PCS) was used. The administration of hyperthermic intra-peritoneal chemotherapy (HIPEC) has been considered another option of regional chemotherapy. The rationale behind is that after exposure of all peritoneal surfaces, the direct contact with hyperthermic chemotherapy generates a higher tissue uptake in residual tumors which is usually limited down to a depth of 1 to 2-mm tumor thickness [32]. In contrast, the depth of penetration with intra-arterial therapy is homogenous throughout the entire tumor tissue, depending on the blood supply of the tumor. Metastases from ovarian cancer are well-vascularized and usually respond even in case of bulky lesions. So far, there have been studies with HIPEC for ovarian cancer in therapy for primary and for recurrent disease [33]. Particularly because of high morbidity and side effects, HIPEC was not recommended as a standard therapy for ovarian cancer [34, 35]. Only recently, however, two randomized studies showed that the addition of HIPEC to cytoreductive surgery resulted in longer survival than surgery alone. Hypoxic abdominal perfusion on the other hand is not associated with relevant toxicity, and quality of life is not impaired. It is not primarily performed in operable cases, appropriate for cytoreduction, but in systemically pretreated, advanced non-operable patients.

Presumably, advances in the treatment of various types of cancer such as ovarian, colorectal, or testicular carcinoma correlate extensively with the chemoresistance of tumor stem cells. While in the recent past, the cure rates of testicular carcinoma and even stage III colorectal carcinoma have increased significantly; the cure rates or clinical progress of ovarian carcinoma have remained unchanged, at between approx. 12% and 14% [36]. This may be related to the low response rate of epithelial ovarian carcinoma stem cells. An increase in overall survival in tumors in general may be due to suppression of the non-stem cell proportion of the tumor. It could also explain why further chemotherapy following recurrences brings about renewed remission and may even prolong life. Due to the existence of the problem of chemoresistant stem cells, these patients have limited therapeutic options and limited prospects for an improvement in prognosis.

Isolated perfusion therapy for bulky disease provides an option to a limited group of patients with advanced bulky disease, to achieve downsizing with operability, in the form of radical cytoreduction. In addition to stage IIIC, this would also apply to stage IV, where conventionally, primary cytoreductive surgery (PCS) has not provided any advantages in the past. The basic principle for increasing the cytostatic effect in the target area is the primary passage through the arterial blood supply with high cytostatic drug concentration, to increase the area under the curve (AUC). A high-concentration passage of the cytostatic in the form of a protracted bolus over 4 to 7 min results in an enhanced first-pass extraction, and based on experience, generate increased tumor necrosis even within the first few days after treatment [37–40]. In order to increase the local cytotoxicity without a significant increase in the overall dosage, the chemotherapeutic agent mitomycin (MMC) and adriamycin (ADM) were selected for perfusion under hypoxic conditions, which, as BA. Teicher described for the first time, produce multiple degrees of their normal cytotoxicity under hypoxic conditions [28]. Thus, the totally administered bone-marrow toxic dose can be kept low. Cisplatin (CDDP) activity is independent of oxygen conditions. Due to the increased cytostatic exposure with isolated perfusion techniques, it is possible to break through chemoresistance in platinum-refractory tumors. The extent to which this applies to tumor stem cells is unknown, but it could apply to initially well-advanced G3 tumors in the case of a few long-term surviving patients following regional chemotherapy. It is particularly remarkable that the survival curves, both in the chemoresistant stage IIIBC as well as IV, do not end abruptly after the median but show a survival rate of 18% and 9% even 3 years after therapy. “A focus on the tail of the survival curves, yields a distinct perspective on the benefit of anticancer therapies and prioritizes therapies with potential to profoundly alter the natural history of disease, even if uncommonly” [41]. This is an important aspect with a selective cohort of patients lifting the “tail” of the survival curve and thus enlarging the area under the curve.

Another important aspect in locally highly concentrated chemotherapy is the lowering of systemic cytostatic exposure by simultaneous chemofiltration [26]. Further improvement in quality of life is achieved in addition to systemic detoxification, through the rapid and high percentage decline in ascites. Seventy three percent of patients reported dramatic relief of their abdominal pain and discomfort. Quality of life should be a priority factor in tumor treatment, especially because newer treatment options, despite some advances, often only lead to slight increases in PFS or overall survival, at the expense of considerable toxicity [42]. As such, the approach of hypoxic abdominal perfusion warrants further investigation in selected patients with ovarian cancer not amenable to surgical resection. In the present situation, there was no control group of heavily pretreated patients available for repeated conventional chemotherapy. Therefore, the pretreated patients receiving isolated perfusion therapy, which is well tolerated due to chemofiltration, serve as their own control. A phase-III study, which investigates HAP versus systemic chemotherapy in non-pretreated patients, could provide information about future treatment options to be adopted.

## Conclusion

Isolated abdominal perfusion under hypoxic conditions for advanced, recurrent, and platinum-refractory ovarian cancer can be an option for multiply pretreated, recurrent ovarian cancer with peritoneal carcinosis, even if drug resistance is existent. Profit in terms of high response rates is achieved by increased tumor toxicity of adriamycin and mitomycin under hypoxic conditions. Due to subsequent chemofiltration, systemic drug exposure is reduced and toxic side effects kept low. Quality of life generally is reported mostly unaffected.

## Abbreviations

*ECOG*: Eastern Cooperative Oncology Group
*CT*: computed tomography
*FIGO*: Fédération Internationale de Gynécologie et d’Obstétrique
*CA*: Carbohydrate antigen
*MMC*: mitomycin C
*CDDP*: Cisplatin
*PFS*: Progression-free survival
*WHO*: World Health Organization
*OS*: Overall survival
*LDH*: Lactate dehydrogenase
*PCS*: Primary cytoreductive surgery
*NACT*: Neoadjuvant chemotherapy
*HIPEC*: Hyperthermic intra-peritoneal chemotherapy
*AUC*: Area under the curve
*TUM*: Tumor marker
*ADM*: Adriamycin
